# Genomic Analysis Illustrated a Single Introduction and Evolution of Israeli Bluetongue Serotype 8 Virus Population 2008–2019

**DOI:** 10.3390/microorganisms9091955

**Published:** 2021-09-14

**Authors:** Natalia Golender, Avi Eldar, Marcelo Ehrlich, Gabriel Kenigswald, Ily Shlamovitz, Boris Even-Tov, Lior Zamir, Eyal Klement, Velizar Bumbarov

**Affiliations:** 1 Division of Virology, Kimron Veterinary Institute, Beit Dagan 5025001, Israel; eldar@moag.gov.il (A.E.); velizarb@moag.gov.il (V.B.); 2 Koret School of Veterinary Medicine, The Robert H. Smith Faculty of Agriculture, Food & Environment, The Hebrew University of Jerusalem, Rehovot 7628604, Israel; 3 The Shmunis School of Biomedicine and Cancer Research, Tel Aviv University, Tel Aviv 6701101, Israel; marceloe@tauex.tau.ac.il; 4 Hachaklait Veterinary Services, Caesarea 3088900, Israel; kenigswald@hak.org.il (G.K.); shlamovitz@hachaklait.co.il (I.S.); 5 Veterinary Servises in the Field, Galil-Golan Veterinary Distinct, Galil-Golan 1231400, Israel; borisev@moag.gov.il (B.E.-T.); liorZ@moag.gov.il (L.Z.)

**Keywords:** bluetongue virus, orbivirus, *Reoviridae*, sequencing, phylogenetic analysis, diagnostics, outbreak, descriptive epidemiology

## Abstract

Outbreaks of the European Bluetongue virus (BTV) serotype 8 (BTV-8), which are characterized by activity cycles separated by years of inactivity, may be influenced by genetic changes of the virus or by herd immunity. BTV activity in Israel is characterized by similar dynamics, but differs from European countries in its vector population, environmental conditions, and lack of cattle vaccination against this serotype. Comparison of these two geographical systems and characterization of their epidemiological connection is therefore of high interest in-order to better understand the factors influencing BTV-8 evolution. BTV-8, closely related to the European strain, was introduced to Israel in 2008. It was at the center of BT outbreaks in 2010 and 2015–2016 and thereafter was lastly isolated in Israel in 2019. We performed genetic analyses of twelve BTV-8 Israeli strains isolated between 2008 and 2019 and compared them with published sequences of BTV-8 isolated in other countries. The analysis revealed a single introduction of BTV-8 into Israel and thereafter extensive occurrence of genomic drifts and multiple reassortments with local BTV strains. Comparison of the Israeli and Cypriot BTV-8 from 2015 to 2016 suggests transmission of the virus between the two countries and a separate and parallel development from European or other Israeli BTV-8 strains. The parallel development of other BTV-8 strains was demonstrated by the identification of the Israeli BTV-8 ISR-1194/1/19 strain, which exhibited common origin with reassorted Israeli BTV-8 strains from 2010 and additional reassortment of seven segments. In order to reveal the source of BTV-8 introduction into Israel we performed BEAST analysis which showed that a probable common ancestor for both European and Israeli BTV-8 presumably existed in 2003–2004. In 2019, a possible new introduction occurred in Israel, where a novel BTV-8 strain was detected, sharing ~95% identity by segments 2 and 6 with Nigerian BTV-8NIG1982/07 and European–Middle Eastern strains. The results of the study indicate that Israel and neighboring countries consist a separate environmental and evolutionary system, distinct from European ones.

## 1. Introduction

Bluetongue (BT) is a non-contagious, vector-borne infectious viral disease of domesticated and wild ruminants caused by bluetongue virus (BTV). It is most commonly observed in sheep and in white-tailed deer, causing severe clinical manifestations and death, especially in naïve animals [1]. BTV is a member of the *Orbivirus* genus within the *Reoviridae*. The double-stranded RNA (dsRNA) genome of BTV is composed of 10 of linear dsRNA segments (Seg-1 to Seg-10) encoding seven structural (VP1 to VP7) and five nonstructural (NS1 to NS5) proteins [2]. Based on Seg-2 gene sequences and virus neutralization tests, currently 35 distinct BTV serotypes have been officially recognized [3]. 

Transmission of most BTV serotypes between mammalian hosts relies on competent blood feeding midges of the *Culicoides* species [1]. Vertical and horizontal transmissions have been described or/and hypothesized, but are considered to be of lesser epidemiological importance [4,5,6,7,8,9].

BTV-8 is endemic in South Africa [10] and there is evidence of its presence in Western Africa (e.g., Nigerian strain NIG1982/07,) [11]). In 2006, BTV-8 emerged in an area between the Netherlands, Belgium, and northern Germany, and then spread throughout the continent and the United Kingdom, causing high mortality in naive sheep flocks and clinical disease in cattle [12,13]. In 2015, after a five-year period with no recorded BTV-8 cases throughout Europe, BTV-8 re-emerged in France, and continued to circulate since then [14]. In late 2018–2020, BTV-8 was reported in Germany, Switzerland, Spain, and Belgium [15,16,17]. In regions more proximal to Israel, BTV-8 was reported (in sheep only) in 2016 in Cyprus [18], and in Turkey the first notification was dated 2018 [19]. In Europe, three BTV serotypes have reappeared (BTV-1, -4, and -8), with consequent disease outbreaks among domestic ruminants. The outbreaks caused by BTV-8 caused significantly more damage and spread to wider territory compared to BTV-1 and -4 [14,15,17,20].

In Israel, BT was first registered in 1944. From 1964 to 2004, five serotypes (BTV-2 -4, -6, -10, and -16) were detected in circulation [21]. From 2006 to present, 12 serotypes (BTV-1, -2, -3, -4, -5, -6, -8, -9, -12, -15, -16, and -24) have been identified in field samples of sick ruminants [22,23,24]. Additionally, two novel serotypes were isolated and identified from commercial sheep pox vaccine [25,26].

BTV-8 was recognized in Israel for the first time at the end of 2008 [22], causing only a limited outbreak in Rosh HaNikra, in northern Israel. However, two years later (2010) it spread throughout Israel and caused outbreaks among sheep, cattle and goats. Twenty-six BTV-8 strains were isolated in 2010 out of a total of 43 isolated BTV strains, being the dominant isolated BTV serotype in that year. Of note, during 2011 and 2013, only a small number of samples from clinically ill animals were identified (by molecular methods only) as positive for BTV-8. In Lebanon, which neighbors Israel and presents geographical continuity with the Israeli northern region, serological investigations suggest BTV-8 circulation during this same period of time [27].

BTV-8 reappeared in Israel in October 2015. During October–December 2015, nine BTV-8 strains were isolated from samples collected from symptomatic animals in two cattle, four sheep, and one goat farms. During the following year, the first outbreak was registered in June 2016 in a sheep farm in northern Israel, further spreading throughout the country among local ruminant population (eleven sheep and fourteen cattle farms); thus, being the dominant BTV serotype that year [23]. Additionally, BTV-8 was isolated from two sheep farms in the West Bank, which exhibited high morbidity and mortality. The predominance of BTV-8 isolation from dead and symptomatic sheep showing typical BT clinical manifestations in Israel in 2016 (i.e., fever, anorexia, excessive salivation, ulcerative, and necrotic lesions and hemorrhages of the oral and nasal mucosa, lameness, facial edema, skin hyperemia, and high mortality) suggested that BTV-8 is the causative agent of these clinical infections. In cattle, illness was observed both in young and milking cows. Lethargy, hypersalivation, nasal discharge, breath abnormalities were the most frequent clinical signs in young cattle, but nasal and oral ulceration and death in rare cases were also observed. In milking cows, the most frequent clinical signs were weakness, recumbency, milk reduction, hypersalivation, tachycardia, and respiratory abnormalities.

The last identified BTV-8 cases occurred in December 2018, and February and October 2019. They included, respectively, positive brain tissue from a single newborn lamb with neural signs, a heifer with limb edema (when BTV-8 probably not associated with this clinical sign), and two sheep showing classical clinical BT manifestation from two different closely situated farms in Israel’s southern district. Surprisingly, in the two last cases, BTV-1 strains were isolated as well; thus, complicating inferences regarding the causative agent of the clinical signs in those sheep.

The present study is aimed at understanding the genetic evolution of BTV-8 in Israel and its comparison with neighboring countries (such as Cyprus) and Europe. We also aimed to understand the time of introduction of the virus into Israel, thus characterizing the epidemiological connection between Israel and the Middle-East and Europe, during the last decade.

## 2. Materials and Methods

### 2.1. Ribonucleic Acid Extraction and Pan-BTV Real-Time Polymerase Chain Reaction (RT-PCR)

We extracted ribonucleic acid (RNA) from the tissue culture supernatant, chicken embryo homogenates and field samples (whole blood, lung, brain, spleen) with: (i) from 2008 to 2012, QIAamp Viral RNA Mini Kit and RNeasy Mini Kit (Qiagen, Hilden, Germany), (ii) from 2012–2017, Invisorb Spin Virus RNA Mini Kit (STRATEC Molecular GmbH, Berlin, Germany) and QIAamp Viral RNA Mini Kit and RNeasy Mini Kit, (iii) from 2017 to 2018, MagMAX™ Pathogen RNA/DNA kit (Thermo Fisher Scientific, Austin, TX, USA), MagVET™ Universal Isolation kit (Thermo Fisher Scientific, Austin, TX, USA), and Invisorb Spin Virus RNA Mini Kit (STRATEC Molecular GmbH, Berlin, Germany), and (iv) since 2019, MagMAX™ CORE Nucleic Acid Purification Kit (Thermo Fisher Scientific, Austin, TX, USA) and Invisorb Spin Virus RNA Mini Kit. Between the years 2008 and 2012, Viral RNA detection was performed by pan-BTV real time polymerase chain reaction (RT-qPCR) using the virotype^®^ BTV pan/8 RT-PCR kits (QIAGEN, Leipzig, Germany) and in-house RT-qPCR based on Seg-1 [28]. Since 2012, VetMAX™ BTV NS3 All Genotypes Kit (Applied Biosystems™, Thermo Fisher Scientific Inc., Lissieu, France) was used for routine BTV screening of field samples. Alternatively, in recent years, the pan-BTV system described by Wernike et al. [29], which is also based on detection of Seg-10 fragment has been employed. In accordance with the instructions of manufacturers of the RT-qPCR kits employed, the cut off for all these methods were Cycle Threshold (Ct) 40.

### 2.2. Type-Specific RT-PCRs

Since 2018, the samples identified as positive for BTV with RT-qPCR with CT values ≤33, were further tested for determining the serotype. Virotype^®^ BTV pan/4 RT-PCR and virotype^®^ BTV pan/8 RT-PCR kits (QIAGEN, Leipzig, Germany) were applied directly to RNA extracted from diagnostic samples to detect and identify BTV-4 and -8 serotypes, respectively. Presence of BTV-3 was tested by an in-house specific RT-qPCR according to the method described by Lorusso et al. [4]. Recently, samples identified as positive by pan-BTV RT-qPCR, were tested by an in-house specific RT-qPCR for BTV-4, -8, and -15 according to the method described by Maan et al. [30]. BTV isolates were tested by in house conventional RT-PCR for BTV-1, and -9 (Table S1), and BTV-2, -5, -6, -12, -16, and -24, as was previously described [23].

### 2.3. Virus Isolation

Since 2010, all BTV RT-qPCR positive samples were inoculated into embryonated chicken eggs (ECE) according to the method described by Komarov and Goldsmit [31] and adopted by Golender et al. [32]. Subsequently, viruses were adapted to Vero (African green monkey kidney epithelial cells) or BHK-21 (baby hamster kidney cells).

### 2.4. Selection of Israeli BTV Strains

Ten BTV-8 strains from 2010 were selected for sequencing of the most frequent reassorted segments (Seg) among Israeli BTV-8: Seg-9 and -10 (according to previously published data on Israeli BTV-8 in GenBank, and BTV-8 strains isolated in 2015–2016, which also exhibited reassortments in these segments). This analysis revealed three reassorted and seven non-reassorted viruses. All three reasserted BTV-8 strains isolated in 2010 were used for further investigations. Additionally, two non-reassorted BTV-8 strains also isolated in 2010 were selected for further characterization and sequencing (Table 1). In 2015 and 2016, two BTV-8 strains were selected from viruses isolated at the beginning or the end of the BT season, one-from sheep and one from cattle. Field samples from all BTV-8 cases in 2018–2019 were used for further characterization of currently circulating BTV-8 Israeli strains.

In order to examine genetic similarity between the reassorted segments and other co-circulating BTV serotypes we analyzed BTV serotypes isolated in the same year or one year after each BTV-8 outbreak (Tables S2 and S3).

### 2.5. Sequencing, BLAST, Pairwise, and Phylogenetic Analyses

cDNA fragments were purified using MEGAquick-spin™ Total Fragment DNA Purification Kit (iNtRON Biotechnology, Gyeonggi-do, Korea) and standard Sanger sequencing was performed on ABI 3730xl DNA Analyzer (Hy Laboratories Ltd., Rehovot, Israel). Nucleotide (nt) sequences were assembled, aligned, and pairwise compared using Geneious (version 9.0.5; Biomatters, Auckland, New Zealand). Phylogenetic trees were constructed using Mega X program [33]. The phylogenetic analyses focused on Israeli BTV-8 strains. For this reason, only a small number of European sequences were used for analysis of homology of genome segments of these viruses.

To gain insight into the phylogeny of the Israeli BTV-8 isolates, we performed BLAST and pairwise analyses of analogous genomic regions. Of note, due to the ~100% identity of all five BTV-8 strains sequenced from Cyprus isolated in 2016, only the CYP2016/01 strain was used for all analyses. Among sequenced non-BTV-8 Israeli strains, only those deemed relevant to BTV-8 strains were used for pairwise and phylogenetic analyses.

### 2.6. BEAST Analysis

The current analysis of BTV-8 employed considerably more nt sequences as compared to the methods described above: 58 concatenated Seg-2 and Seg-6 sequences including 12 Israeli and 46 European strains were used for BEAST v1.10.4 version of the program [34]. We chose to perform this analysis only on Seg-2 and Seg-6 as the frequent reassortment events which occurred in Israeli BTV-8 strains during the study period prevented evolutionary inference with other segments. jModelTest v2.1.10 [35] was used for choosing an appropriate model for BEAST analysis. The program showed HKY strict model as the best model for the analysis. Nevertheless, in addition to the model chosen by the program, several different evolutionary models, including HKY and GTR, including the strict and uncorrelated relaxed clock, were run for analyses for predicting the common ancestor and substitution rates. All sequences were analyzed together without separation of the 1st and 2nd European outbreaks or separation of Israeli (2010) and Israeli–Cyprian outbreaks (2015–2016).

## 3. Results

### 3.1. Laboratory Diagnosis Tests of BTV Detection in 2008–2017

Due to inaccessibility regarding data on molecular BTV laboratory diagnosis in 2008–2010, Table 1 only shows information on virus isolation in 2008–2010, reporting thus on when BTV-8 were successfully isolated. Given that no BTV-8 was isolated in 2011–2014, and due to the lack of systematic RT-qPCR screening tests, which could allow estimating BTV-8 prevalence in received field samples, no data from these years are shown. Table 1 also summarizes data on molecular detection of all BTV cases in field samples since 2015, and successful virus isolations.

### 3.2. BTV Detection by Pan-BTV RT-qPCR from Field Samples in 2015–2019

Data regarding the presence of BTV in the samples examined by BTV RT-qPCR between 2015 and 2019 are shown in Table 1. A total of 3997 samples were tested for the presence of BTV. Of these, 1005 were found positive by BTV RT-qPCR. Of the 603 samples collected in 2015, 143 were found positive for BTV, 228 of the 669 collected in 2016, 168 of the 744 collected in 2017, 271 of the 1022 collected in 2018 [23], and 195 of the 959 collected in 2019.

### 3.3. Serotype Specific RT-qPCR Result of Tested BTV-Positive Tested in 2019

A total of 188 samples collected in 2019, in which the Ct value was ≤33, were tested by BTV-3, -4, and -8 specific RT-qPCRs. Details on these results obtained from the tested samples are shown in Table 2. Four whole blood samples and one mixed spleen and lung sample from cattle and two whole blood samples from sheep had BTV-3 and -4 double infection in 2019. In two sheep whole blood samples, BTV-1 and -8 were identified (Table 2). However, due to incomplete molecular investigation on other recognized BTV serotypes, we cannot provide the full data on both specific serotype incidence and other possible mixed BTV infections in the current article.

### 3.4. The Last Detected Cases of BTV-8

Screening of positive BTV samples (Ct value of ≤33) for BTV-8 Seg-2began in 2018. No BTV-8 were identified by this method during this year [23]. Nevertheless, the brain of one newborn sheep exhibiting neural signs was positive in RT-qPCR and sequencing analyses of Seg-5, indicating its probable belonging to a BTV-8 serotype. This case, as well as the fact that the next identification of BTV-8 occurred in February 2019, are in accord with a low circulation of BTV-8 during the second half of 2018. BTV-8 was last detected at the end of 2019, from two sheep co-infected with BTV-1. Unfortunately, attempts of BTV-8 isolation from these field samples were unsuccessful. Data on all BTV-8 Israeli strains used in the study is shown in the Table S2. As the current study focuses on BTV-8 we have compiled, Table 3 presents information on the last confirmed BTV-8 cases (2018–2019).

### 3.5. Virus Isolation (VI)

Data on virus isolation is summarized in Table 1. From 2015 to 2019, of the 1005 samples positive by BTV RT-qPCR and inoculated into ECE; 196 BTV strains were isolated. In five occasions, simultaneous BTV-3 and BTV-4 were isolated (*n* = 4 in 2018 and *n* = 1 in 2019), while one simultaneous isolation of BTV-3 and BTV-8occured in 2016 [23].

### 3.6. Sequencing

Length of fragments and accession number (acc. num.) of all sequenced Israeli BTV-8, and other Israeli serotypes circulated in 2011–2019 in Israel, and not sequenced before, are summarized in Table S3.

### 3.7. BLAST, Pairwise, Phylogenetic and BEAST Analyses

**Figure 1:**
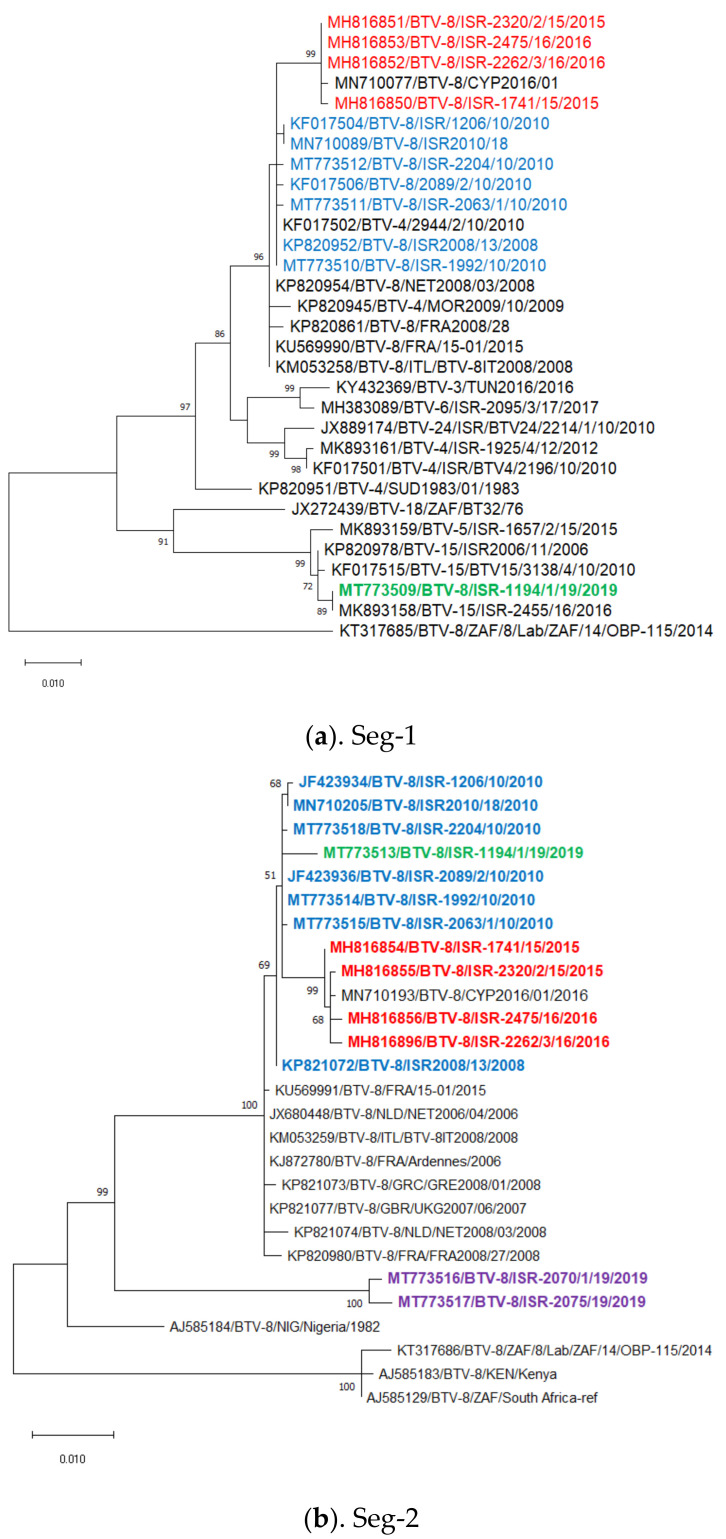

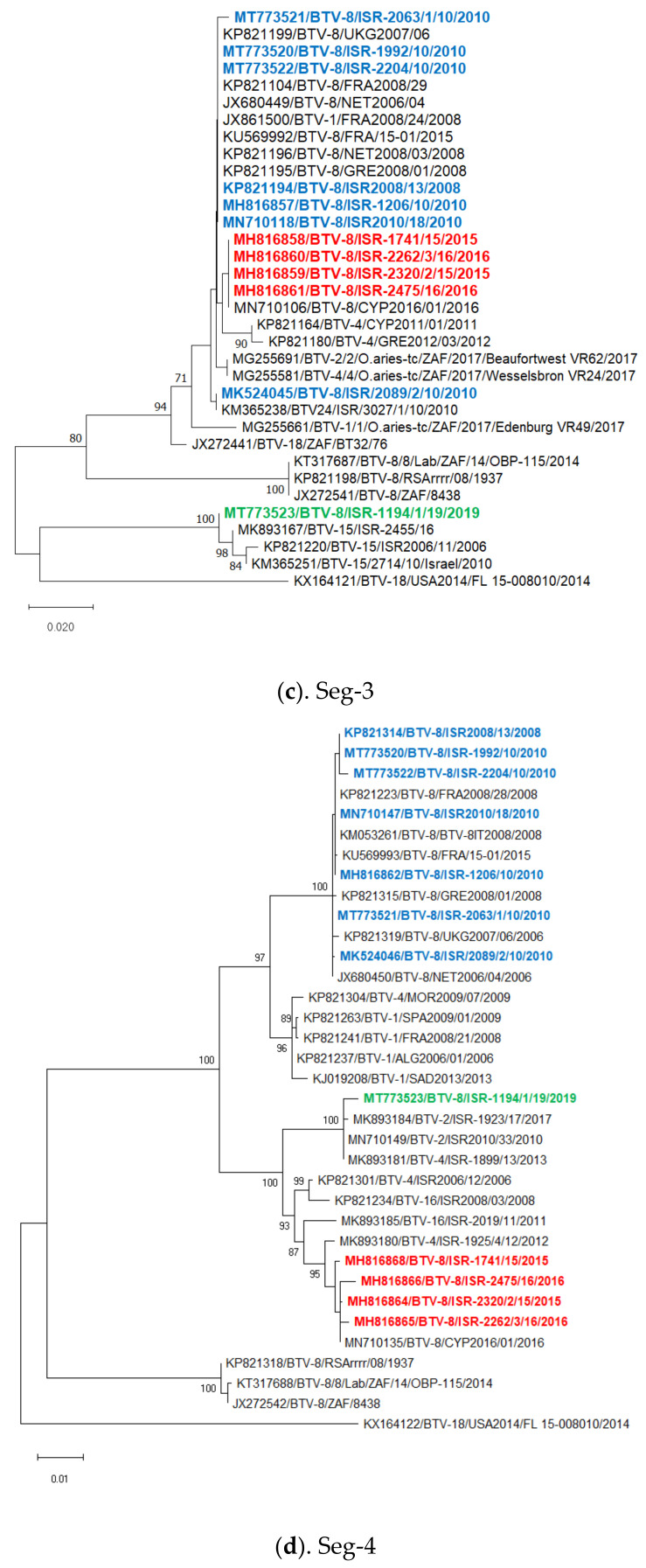

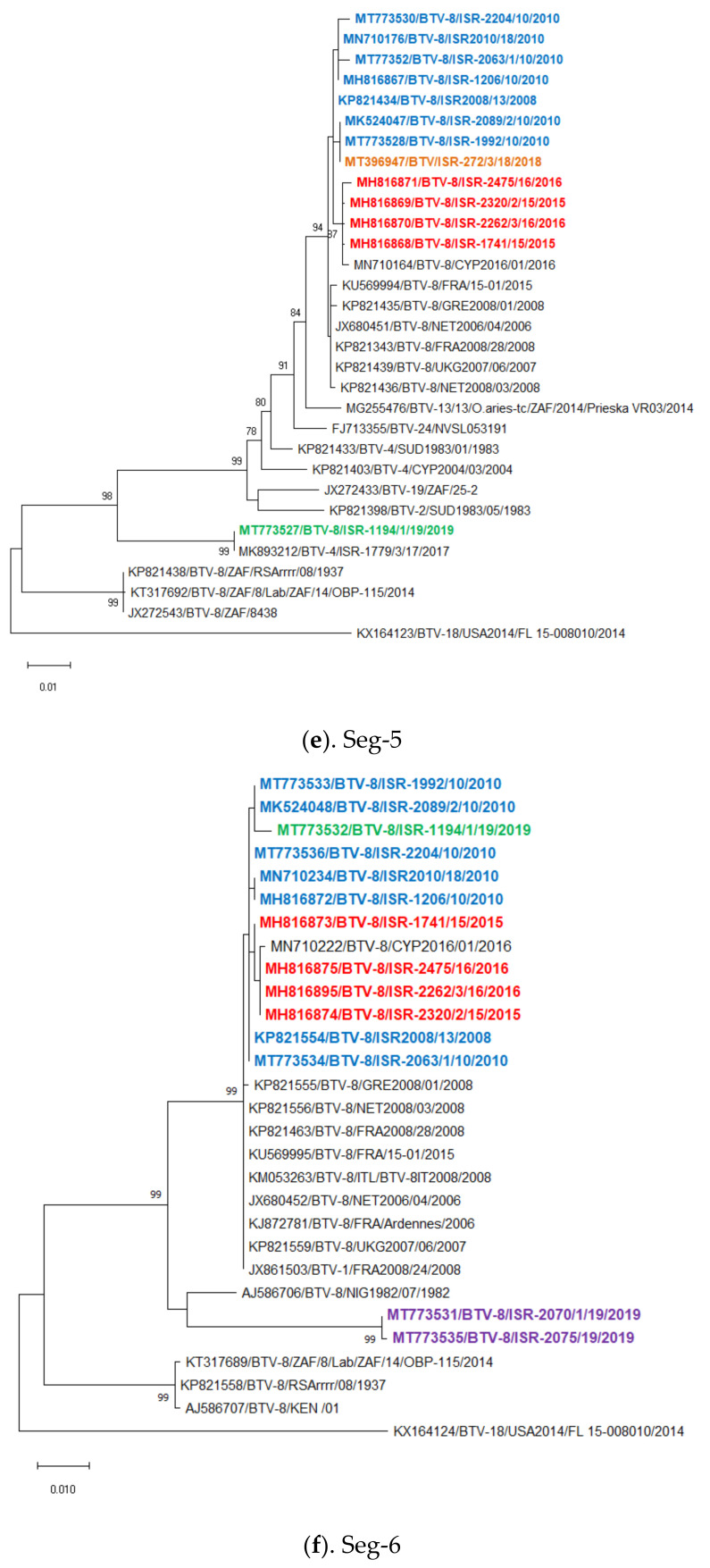

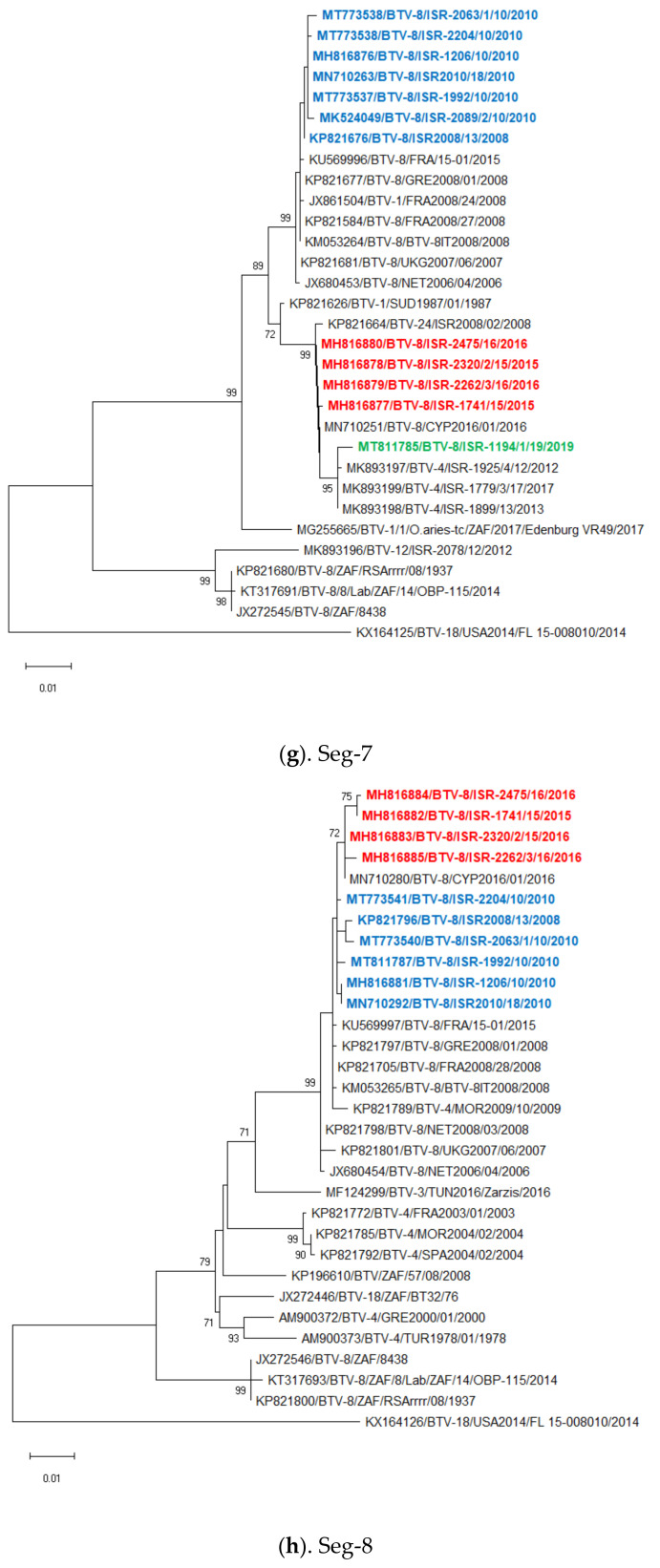

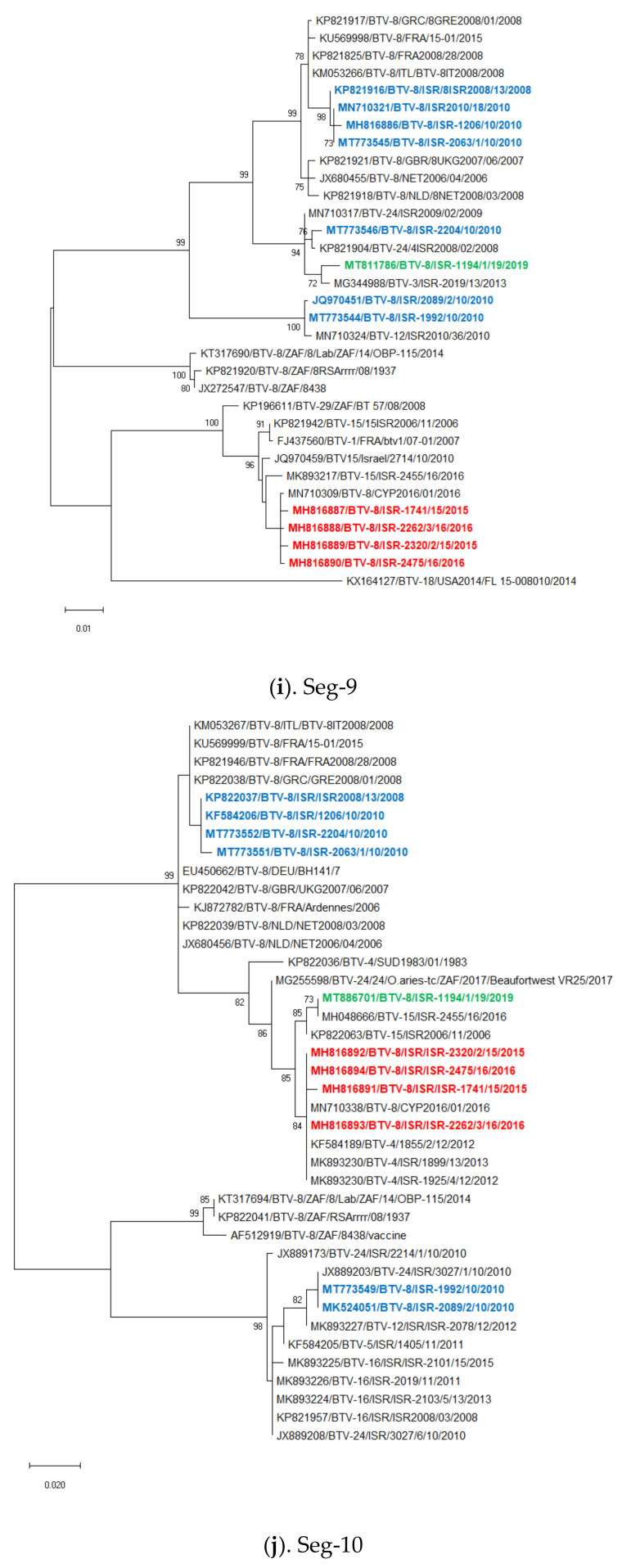
Phylogenetic tree of Israeli BTV-8 strain 2008–2019 and related BTV. (**a**) represents segment 1; (**b**)—segment 2; (**c**)—segment 3; (**d**)—segment 4; (**e**)—segment 5; (**f**)—segment 6; (**g**)—segment 7; (**h**)—segment 8; (**i**)—segment 9; (**j**)—segment 10. The phylogeny was inferred using the maximum likelihood method and the Tamura–Nei model method. The percentage of replicate trees in which the associated taxa clustered together in the bootstrap test (1000 replicates) are shown next to the branches. Viruses were identified by accession number/serotype/location/isolate/year. Israeli BTV-8 strains isolated in 2008–2010 are shown in blue, in 2015–2016—red, in 2018—orange, in February 2019—green, and in October 2019—purple.

The same sequences were used for pairwise and phylogenetic analyses (their acc. num. are presented in the phylogenetic trees). Phylogenetic analysis revealed only slight differences between the Israeli prototype BTV-8 ISR2008/13 strain and the European BTV-8 strains isolated in 2006–2009, indicating their common origin. The phylogenetic analysis indicates that the Israeli BTV-8 isolated in 2010 probably originated from a common ancestor with the Israeli BTV-8 prototype strain (ISR2008/13) (nt identity—99.48–100%). Most of the Israeli BTV-8 isolates were different from the ISR2008/13 strain by only point mutations, suggesting genetic drift as the main mechanism of the change. Concerning reassortments, only one out of ten tested BTV-8 isolated in 2010 had a single reassortment of Seg-9 (ISR-2204/10 strain, isolated from sheep from the Israeli southern distinct), while another two isolates (strains ISR-1992/10 from cattle and ISR-2089/2/10 from sheep, both from the northern part of Israel) had two reassorted segments: Seg-9 and 10. Notably, the reassorted Seg-9 differed in origin between the single and double reassorted strains. All reassorted segments of these strains probably originated from viruses circulating in Israel during 2008–2010. These reassorted strains where isolated from field samples collected from symptomatic animals in August–September 2010. Genetic analyses (comprising Seg-1, -2, -3, -5, -6, -8) of Israeli and Cypriot strains isolated in 2015–2016 revealed segregation of Israeli–Cypriot strains into a separate group, different from the previously circulating Israeli BTV-8 strains (nt identity with the Israeli viruses isolated in 2008–2010 was 99.02–99.81%). When considering additional segments in this analysis (Seg-4, -7, -9 and -10), we propose that their origin stems from reassortments with local BTV strains, which circulated in this region in the period of 2008–2013 (Figure 1d,g,i,j). Sequence analysis of the Israeli and Cypriot BTV-8 strains, suggests transmission of these strains between the two countries, parallelly and separately from the current European strains, and even from other Israeli BTV-8 strains. Interestingly, analysis of segments 2 and 6 revealed the probable common origin of the ISR-1194/1/19 strain and the strains isolated in 2010 (Figure 1b,f). However, analysis of internal genes of the last Israeli strain revealed reassortments with more recent (circulating since 2013) and local non-BTV-8 viruses, instead of sequences originating from the Israeli–Cypriot strains. Table 4 summarizes the information on the mentioned reassortments. All other phylogenetic trees are shown in Figure 1c,e,h.

Genetic, phylogenetic and pairwise analysis of Seg-2 and -6 of recently identified BTV-8 from sheep in October 2019 showed about 95% identity both with European–Israeli strains and the Nigerian NIG1982/07 strain, suggesting introduction of a novel BTV-8 strain into Israel.

### 3.8. Phylogenetic Analysis BEAST Analyses of Concatenated Sequences

**Figure 2:**
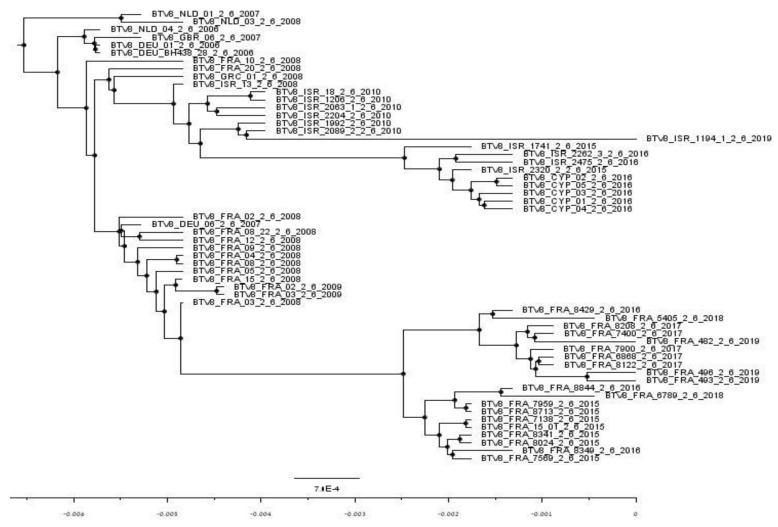
Phylogenetic tree of Israeli and European BTV-8 2006–2019 concatenated by segments 2 and 6 having the common ancestor (4571 positions in the final dataset). The phylogenetic tree was created using FigTree v 1.4.4 [34]. The scale bar showed nucleotide substitution per size. Presumable common ancestors are signed by black dots.

BEAST analysis illustrated the probable common ancestor of the European and Israel BTV-8 dated June 2003–January 2004. It is difficult to evaluate the speed of evolution of Israeli viruses due to the small number of sequenced virus isolates. Nevertheless, the parallel evolution of European and Israeli strains is evident from this analysis. Moreover, several different constellations of BTV-8 have probably been developing in parallel in Israel/Middle-East: one that caused an outbreak in 2015–2016 in Israel and Cyprus, and at least one more that developed from the common ancestor of the double reassorted strains (ISR-1992/10 and ISR-2089/2/10) detected in August–September 2010. Notably, these strains were sampled from the same geographic area (northern part of Israel) as the last BTV-8, which was found, sampled in February 2019 (ISR-1194/1/19). Considering strains from the Middle-East outbreak, Israeli BTV-8 strains were the ancestors to Cyprian BTV-8 strains in 2016. Summarizing data from all phylogenetic analyses of nt sequences of Seg-2 and -6 (concatenated and separate analyses), we can presume the common origin of Israeli and European BTV-8 (Figure 2). However, it is still impossible to estimate the real origin and the way of introduction of closely related BTV-8 in both Israel and Europe.

**Figure 3:**
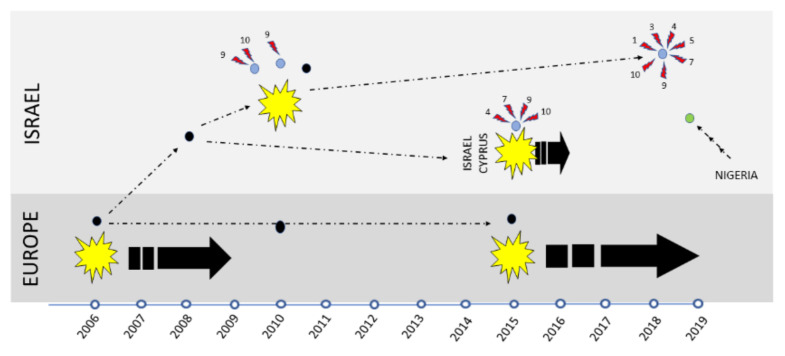
Schematic date-related evolution and introduction events of BTV-8 in Israel and Europe in 2008–2019. Black spots—prototype and closely related to European BTV-8 strains. Bluish spots—reassorted strains. Red lightning—reassorted segments. Yellow stars—epidemics caused by strains schematically shown above the star. Black thick arrows—illustrate the durations of the epidemics. Black thin dotted arrows—depict the probable relations of viruses from the previous to the next outbreak. All schematic illustrations are shown according to date-frame (shown at the bottom of the figure).

Schematic date-related illustration of introduction and evolution events occurred with Israeli BTV-8 strains are shown in Figure 3.

## 4. Discussion

In this study, we compared and contrasted the genetic composition of BTV-8 strains isolated from outbreaks and cases, which occurred in Israel between 2008 and 2019, with the objective of identifying potential sources for their sequence variability. It was previously suggested that the source of introduction of BTV-8 to Israel was the importation of fattening calves from Europe [22]. The only BTV-8 strain isolated in close temporal proximity to this presumed instance of introduction, ISR2008/13, was isolated from a milking cow from the north of Israel, in a location situated near the Israeli–Lebanon border. Thus, additional possible routes of introduction should be considered. Among these are the wind-borne migration of blood sucking midges or the trade and subsequent movement of infected animals (as described previously for the introduction of bovine ephemeral fever into Israel by Aziz-Boaron et al., [36]). Analysis of the strains isolated in recent years suggests that no further exogenous introduction of BTV-8 occurred until 2019. If so, the viruses isolated in Israel or Cyprus, subsequent to the initial outbreak of 2008–2010 (e.g., in the outbreak of 2015–2016) must have originated from the development of Israeli BTV-8 strains introduced in 2008–2010. Such a scenario would suggest a parallel (i.e., separate) development of BTV-8 strains in Europe and Israel.

Given the segmented nature of the BTV genome, the reassortment of genetic segments functions as a main source of change in BTV evolution. In this context, it has been shown previously that the genetic exchange of any of 10 segments is equally likely to occur [37]. In regions, where multiple BTV serotypes are co-circulating, this phenomenon may be considerably frequent. An example of this phenomenon are the reassorted BTV-1 and BTV-4 strains, which circulated in Morocco and France and can also be observed in the phylogenetic trees presented in the current article (Figure 1b,h, illustrating Seg-1 and-8, BTV-4, MOR2009/10 strain; Figure 1c,f,g illustrating Seg-3, -6, and -7 of BTV-1, FRA2008/24 strain). Similarly, in Israel, where multiple distinct BTV serotypes are co-circulating, the theoretical probability for reassortment is high [23,24,38]. Curiously, the exchange of genome segments does not appear to be symmetrical among serotypes. While BTV-8 strains accepting gene segments from circulating Israeli BTV serotypes were abundant, we detected only two occasions in which the opposite direction of reassortment was observed: BTV-4 2944/2/10 strain segment 1 (Figure 1a), and BTV-24 3027/1/10 strain, where Seg-3 appears to be contributed by BTV-8 (Figure 1c). Notably, both these strains were obtained from the sheep farms where several serotypes were circulating simultaneously (BTV-4, -8 and -24) and double/triple infection of the same animal was confirmed (an official report from Pirbright reference laboratory, 2011). Nevertheless, no evidence of continuous circulation of such reassorted strains was observed. Generally, the high frequency of reassortment of local BTV-8 strains can be explained by the frequent co-infection of several BTV serotypes of the same host. The outcome of this co-infection process, in which many different reassortment combinations can be generated, but only some prosper, may testify to the adaptation of prospering strains to the environmental conditions or vector abundance in Israel.

Already in 2010 (two years after the supposed introduction in 2008), we could detect three instances of reassorted BTV-8 strains (in addition to non-reassorted strains): ISR-2204/10, where only Seg-9 was reassorted: ISR-2089/2/10 and ISR-1992/10, where reassortments of Segs-9 and -10 were identified. Notably, these reassortments appear to have occurred with local strains. Our phylogenetic analysis of recently isolated circulating BTV-8 strains suggests that virus of this serotype continued to reassort with local strains. For example, in the ISR-1194/1/19 strain, seven of nine sequenced segments were reassorted, while only Seg-2 and -6, which encode the outer capsid BTV proteins (VP2 and VP5) were of Israeli BTV-8 origin, probably from the same ancestor as two reassorted strains from the same geographic area: ISR-1992/10 and ISR-2089/2/10.

The occurrence of multiple of outbreaks only two years after the introduction of BTV-8 to Israel in 2008 raises questions regarding their adaptation processes. The identification of the prevalent BTV-8 strain in each outbreak may shed light on the genetic mechanisms enabling a specific BTV-8 to spread more efficiently than their counterparts, causing in this manner an epidemic.

Israeli BTV-8 isolated in 2015–2016 are the probable ancestors to closely related Cypriot BTV-8 strains isolated in 2016, as they have the same four reassorted segments and as revealed from and BEAST analysis of concatenated sequences. While separated by sea, and thus lacking territorial continuity, it should be noted that atmospheric transport between Israel and Cyprus is observed (mainly in summer) [39], thus providing a possible mechanism for the sharing of serotype/strain prevalence in these two countries.

Ceaseless annual introduction of orbiviruses into Israel commonly leads to short outbreaks, as seen for EHDV-1, -6, -7, and BTV-6 [32,40]. Some, however, lead to long-lasting outbreaks and to the occurrence of novel endemic BTV serotypes in Israel. Examples of this latter phenomenon include BTV-3 [23], and the reappearance of some serotypes after periods of 4-6 years silence (e.g., BTV-5, BTV-15 and BTV-8). Most recently, in 2019, at least two novel serotypes invaded Israel (BTV-1 and -9 [3]) along with an introduction of novel BTV-8 strains. Except for BTV-8 strains, all other BTV serotypes do not have very close ancestors with European strains, indicating different routes of introduction and different evolution. Notably, all Israeli BTV strains were most closely related to African strains. However, we cannot be sure of their route of introduction or true origin, as there are many “white spots on the map” regarding circulating strains in regions neighboring Israel (e.g., additional Middle-East or Asian countries).

## 5. Conclusions

The scenario we propose for the evolution of BTV-8 in Israel is based on the development of local BTV-8 population through both genomic drift and reassortment; essentially independent from the parallel evolution of European BTV-8 strains. In particular, we have identified a novel branch of Israeli–Cypriot BTV-8 strains, which separated from the prototype Israeli BTV-8 strains. Concomitantly to this branching event, additional BTV-8 strains originated from the same Israeli BTV-8 prototype have also developed. Our analysis also spans very recent events (2019) where we identified serotypes that are novel in Israel (BTV-1 and -9), in addition to novel BTV-8 strains. Altogether the analysis shows that in the Middle-East BTV circulation is independent from Europe.

Nevertheless, the continuous invasion of BTV and other orbiviruses to Israel constitute only a part of a much wider environment–host–vector–pathogen ecosystem, which includes many different viruses. For example, the Shuni virus, which belongs to family *Peribunyaviridae*, recently invaded Israel, and is associated with abortion events, malformations, and even neural signs and fatalities in young cattle [41]. This high rate of newly emerging *Culicoides*-borne viruses in Israel is probably related to its climatic conditions, which are ideal for the development of *C. imicola* and other competent *Culicoides* vectors. The ongoing global warming may enhance such incursions of new viruses into new locations. The location of Israel, between three continents, make it an ideal sentinel for sampling, observation, and investigation of the movement of viruses between countries and continents, thus enabling early detection of such incursions.

## Figures and Tables

**Table 1:** Information on BTV isolation and pan-BTV RT-qPCR from different kinds of domestic and wild/zoo ill or dead animals during the years, when BTV-8 were isolated.

Species		Cattle			Sheep			Goat			Wild Ruminants					
Year/Organ		w.b.	s/l	a.f	.w.b.	s/l	a.f.	w.b.	s/l	a.f.	w.b.	s/l	a.f.	Total Samples	Total Vi	Source
2008	No. of isolated BTV-4	2										1			3	current study
	No. of isolated BTV-8	1													1	
	No. of isolated BTV-16	1							1						2	
	No. of isolated BTV-24	2			3	3									8	
	No. of untyped isolated BTV	4	1		1	2						1			9	
2010	No. of isolated BTV-2	1										1			2	current study
	No. of isolated BTV-4	4			1	1									6	
	No. of isolated BTV-8	10	3		9	2		1	1						26	
	No. of isolated BTV-12	2													2	
	No. of isolated BTV-15	2													2	
	No. of isolated BTV-24	1			5										6	
2015	No. of tested samples	470	12	2	50	20	6	17	8	1	9	8	0	603		current study
	No. of pos. samples	98	3	1	25	5	3	5	2	0	1	0		143		
	No. of isolated BTV-4	2			1			1							4	
	No. of isolated BTV-5	5			2										7	
	No. of isolated BTV-8	6			2			1							9	
	No. of isolated BTV-16	1			1										2	
2016	No. of tested samples	484	41	10	73	20	18	1	4	5	0	11	2	669		[23]
	No. of pos. samples	159	13	1	49	5	0	0	1	0	0	0	0	228		
	No. of isolated BTV-2				3										3	
	No. of isolated BTV-3				3										3	
	No. of isolated BTV-4	1													1	
	No. of isolated BTV-8	24	1		26										51	
	No. of isolated BTV-15	1													1	
2017	No. of tested samples	402	35	7	173	41	10	9	24	0	17	23	3	744		[24]
	No. of pos. samples	93	6	0	51	9	0	0	6	0	1	2	0	168		
	No. of isolated BTV-2	1													1	
	No. of isolated BTV-3				4										4	
	No. of isolated BTV-4	1			12										13	
	No. of isolated BTV-6	6			10										16	
	No. of isolated BTV-15	6													6	
2018	No. of tested samples	629	18	53	72	32	156	8	21	14	4	13	3	1022		[23]
	No. of pos. samples	217	6	1	36	0	6	3	2	0	0	0	0	271		
	No. of isolated BTV-2	1													1	
	No. of isolated BTV-3	1			6			1							8	
	No. of isolated BTV-4	1			9										10	
	No. of isolated BTV-6				2										2	
	No. of isolated BTV-15	9													9	
2019	No. of tested samples	490	45	87	109	51	98	7	5	13	23	26	5	959		current study
	No. of pos. samples	106	8	1	52	17	7	4	0	0	0	0	0	195		
	No. of isolated BTV-1	3			2										5	
	No. of isolated BTV-3	5	1		2										8	
	No. of isolated BTV-4	1			6										7	
	No. of isolated BTV-9	7			17	1									25	

No.—number; w.b—whole blood samples; s/l—spleen or lung samples; a.f.—aborted fetus; VI—virus isolation.

**Table 2:** Information on BTV-positive field samples tested in serotype-specific RT-qPCRs in 2019.

Species		Cattle			Sheep			Goat			
Specific RT-qPCR/Organ		w.b.	s/l.	a.f.	w.b.	s./l.	a.f.	w.b.	s./l.	Total	Source
BTV-3	No. of tested samples	103	8	0	51	15	7 ^1^	4	0	188	current study
	No. of positive samples	19	2	0	18	3	1 ^2^	0	0	43	
BTV-4	No. of tested samples	103	8	0	51	15	7	4	0	188	
	No. of positive samples	12	3	0	16	2	0	1	0	34	
BTV-8	No. of tested samples	103	8	0	51	15	7	4	0	188	
	No. of positive samples	1	0	0	2	0	0	0	0	3	

^1^—seven samples were collected from 5 abortion cases including five placentas, two brain and one lung samples. ^2^—BTV-3 positive case was positive in conventional BTV-3-specific RT-PCR and confirmed by sequencing.

**Table 3:** Information on the last confirmed BTV-8 cases (2018–2019) among routine diagnostics of field samples.

	RT-qPCR Ct Value			
Strain	Pan-BTV	BTV-8	Additional Diagnosis	Sequencing
ISR-272/3/18	36.33	NA	no	BTV-8 (Seg-5)
ISR-1194/1/19	33.65	31.74	no	BTV-8 (Seg-1–7, 9,10)
ISR-2070/1/19	17.13	33.4	BTV-1 ^1^ PCR, BTV-1 VI	BTV-1 (Seg-2), BTV-8 (Seg-2 and 6)
ISR-2075/19	20.92	27.2	BTV-1 ^1^ PCR, BTV-1 VI	BTV-1 (Seg-2), BTV-8 (Seg-2 and 6)

^1^—RNA from the whole blood samples were tested by conventional RT-PCR with BTV-1 specific primers, found positive and confirmed by sequencing analysis. VI—virus isolation. Seg-segment.

**Table 4:** Summary information of the analyzed Israeli BTV-8 strains compared with the prototype ISR2008/13 strain for the reassorted segments and their percentages of gemology with the prototype strain.

Strain	Seg-1	Seg-2	Seg-3	Seg-4	Seg-5	Seg-6	Seg-7	Seg-8	Seg-9	Seg-10
ISR-1206/10	H	H	H	H	H	H	H	H	H	H
	99.90	99.90	99.82	100	99.92	99.81	99.91	99.91	99.69	99.87
ISR-2063/1/10	H	H	H	H	H	H	H	H	H	H
	99.88	99.86	99.80	100	99.58	99.94	99.70	99.80	99.89	99.75
ISR-2089/2/10	H	H	H	H	H	H	H	H	R ^1^	R ^1^
	99.84	99.79	99.78	99.90	99.84	99.93	99.85	99.79	93.43	83.31
ISR-1992/10	H	H	H	H	H	H	H	H	R ^1^	R ^1^
	100	99.93	99.90	100	99.86	99.87	99.90	99.81	93.02	82.90
ISR-2204/10	H	H	H	H	H	H	H	H	R ^2^	H
	99.86	99.85	100	99.80	99.59	99.94	99.80	99.91	96.22	99.87
ISR-1741/15	H	H	H	R ^1^	H	H	R ^1^	H	R ^3^	R ^2^
	99.70	99.69	99.67	95.01	99.72	99.94	98.16	99.72	90.44	95.18
ISR-2320/2/15	H	H	H	R ^1^	H	H	R ^1^	H	R ^3^	R ^2^
	99.72	99.59	99.75	95.03	99.72	99.88	98.27	99.81	89.86	95.30
ISR-2262/3/16	H	H	H	R ^1^	H	H	R ^1^	H	R ^3^	R ^2^
	99.67	99.42	99.67	94.97	99.72	99.88	98.27	99.54	90.31	95.56
ISR-2475/16	H	H	H	R ^1^	H	H	R ^1^	H	R ^3^	R ^3^
	99.70	99.49	99.75	95.22	99.66	99.87	98.27	99.62	89.94	95.49
ISR-1194/1/19	R ^1^	H	R ^1^	R ^2^	R ^1^	H	R ^2^	NA	R ^4^	R ^4^
	94.42	99.53	87.22	94.52	92.38	99.43	97.50	NA	97.18	94.05

H—homologous segment, where only point mutations were observed; R—reassorted segment; NA—not amplified; attempts to sequence these segments were unsuccessful. 1–4-number of different reassortment variant of the specific compared to previously isolated BTV-8 strains (see Figure 1).

## Data Availability

Not applicable.

## Supplementary Materials

The following are available online at https://www.mdpi.com/article/10.3390/microorganisms9091955/s1, Table S1. List of primers used for BTV-1 and BTV-9 serotyping and BTV-8 for sequencing of outer genes. Table S2. Information about selected for characterization Israeli BTV-8 strains. Table S3. List of sequenced Israeli BTV strains for the present study.
