# Supplementary material for: Genomic Analysis Illustrated a Single Introduction and Evolution of Israeli Bluetongue Serotype 8 Virus Population 2008–2019
Source: Microorganisms. 2021 Sep 14;9(9):1955. doi: 10.3390/microorganisms9091955 (PMC8470199; doi:10.3390/microorganisms9091955)
Supplement: Supplementary file 1 [file microorganisms-09-01955-s001.zip › Table S1-primers.pdf]

Table S1. List of primers used for BTV-1 and BTV-9 serotyping and BTV-8 for sequencing of outer genes.

Serotype/ Segment	Name	Sequence ('5–3')	Length of Product	Annealing t°C	Source
1/2	1VP2-1341F	CAAGGGAACCCATGTGATT	694	54	current study
	1VP2-2012R	CTTGAGAGATATCCACAATGCTC			
1/2	1VP2-1769F	ACAGGCACAGTCCACTTACG	856	54	current study
	1VP2-2606R	TTTGTTCGGATGTGTGATC			
8/2	8P2-6F	AAT AGC GTC GCG ATG GAG GAG C	590	54	current study
	8P2-574R	AAT GTA TAC GCC GCC TCT TGT G			
8/2	8P2-34F	TTC CGA TTT ATA CGA ATG TAT TCC C	762	54	[24]
	8P2-772R	TCA AAG CGG TTA TTT CCT CTT GTA			
8/2	8P2-584F	GGC GTA TAC ATT GAA ACC CAC A	823	54	current study
	8P2-1382R	CGC CTC ATG AAA CTT GGT GGT AAA T			
8/2	8P2-1298F	TTG GGA GTA TAA GTT GCT TAA TGA A	930	54	current study
	8P2-2205R	GCG ATA TCT TGA TTT ATC TTT GC			
8/2	8P2-1958F	TGA AGT AAC GTT TGA ACA CCC AAC A	681	54	current study
	8P2-2916R	TAA GTT GAT AGC GCG CGA GCT CT			
8/6	8VP5-24F	GAAGATGGGGAAAATCATAAA	935	54	current study
	8VP5-939R	CGATGGCTCTATTCTTTGAT			
8/6	8VP5-851F	CAC CGA AGA TTG AGC CTA CGA	786	55	current study
	8VP5-1616R	TAA GTG GAA AGC GGT GGC TCC			
8/6	8VP5-1F	GTTAAAAAAGCGATCGCTY	1637	54	current study
	8VP5-1618R	GTAAGTGGAAAGCGGTGGCT			
9/2	9VP2-2598F	CCG ATT AAG TGY TTA ACT TTG A	299	54	current study
	9VP2-2896R	TGT CAC TGG GAC TAT ACR TTC A			
