# Supplementary material for: Genomic Analysis Illustrated a Single Introduction and Evolution of Israeli Bluetongue Serotype 8 Virus Population 2008–2019
Source: Microorganisms. 2021 Sep 14;9(9):1955. doi: 10.3390/microorganisms9091955 (PMC8470199; doi:10.3390/microorganisms9091955)
Supplement: Supplementary file 1 [file microorganisms-09-01955-s001.zip › Table S2-data on BTV-8 strains.pdf]

Table S2. Information about selected for characterization Israeli BTV-8 strains.

Strain	Date of Sampling	Species	Age Category	Geographic Place	Source
ISR2008/13	Nov-2008	cattle	adult	Kfar Rosh Ha Nikra, Coastal Area, Northern Distinct	GenBank
1206/10	16-Feb-2010	cattle	adult	Moshav Peduim, Negev Desert, Southern Distinct	current work
ISR-2089/2/10	Aug-2010	sheep	adult	Yokneam-Illit, Lower Galilee, Northern Distinct	current work
ISR-2063/1/10	28-Aug-2010	cattle	adult	Kfar Shmuel, Central Dictinct	current work
ISR-1992/10	8-Sep-2010	cattle	adult	Moshav Regba, Coastal Area, Northern Distinct	current work
ISR-2120/10	5-Sep-2010	cattle	adult	Kfar Blum, Golan Heght, Northern Distinct	current work
ISR-2204/10	20-Sep-2010	sheep	adult	Rahat, Negev Desert, Southern Distinct	current work
ISR-1741/15	10-Okt-2015	cattle	calf	Kitutz Or HaNer, Negev Desert, Southern Distinct	current work
ISR-2320/2/15	10-Dec-2015	sheep	adult	Paran, Arabah valley, Southern Distinct	current work
ISR-2262/3/16	13-Oct-2016	sheep	adult	Kfar Mikne Dekel, Negev Desert, Southern Distinct	current work
ISR-2475/16	20-Dec-2016	cattle	calf	Moshav Beit Hillel, Upper Galilee, Northern Distinct	current work
ISR-272/3/18	12-Dec-2018	sheep	newborn	Dimona, Negev Desert, Southern Distinct	current work
ISR-1194/1/19	07-Feb-2019	cattle	adult	Kibutz Afikim, Jordan Valley, Northern Distinct	current work
ISR-2070/1/19	23-Oct-2019	sheep	adult	Kfar Maimun, Negev Desert, Southern Distinct	current work
ISR-2075/19	29-Oct-2019	sheep	adult	Moshav Tkuma, Negev Desert, Southern Distinct	current work
