# Supplementary material for: Genomic Analysis Illustrated a Single Introduction and Evolution of Israeli Bluetongue Serotype 8 Virus Population 2008–2019
Source: Microorganisms. 2021 Sep 14;9(9):1955. doi: 10.3390/microorganisms9091955 (PMC8470199; doi:10.3390/microorganisms9091955)
Supplement: Supplementary file 1 [file microorganisms-09-01955-s001.zip › Table S3-seq.pdf]

Table S3. List of sequenced Israeli BTV strains for the present study

serotype	host/year	segment/ length<br>strain	1/3944	2/2926-2939	3/2772	4/1981	5/1774-1776	6/1637-1645	7/1156	8/1125-1131	9/1047-1050	10/822
8	cattle/2015	ISR-1741/15	MH816850 1-3944	MH816854 1-2938	MH816858 4-2767	MH816863 33-1894	MH816868 1-1774	MH816873 1-1637	MH816877 18-1103	MH816882 27-1096	MH816887 1-971	MH816891 60-764
8	sheep/2015	ISR-2320/2/15	MH816851 1-3944	MH816855 1-2938	MH816859 3-2768	MH816864 1-1870	MH816869 1-1775	MH816874 1-1637	MH816878 8-1105	MH816883 29-1092	MH816889 32-986	MH816892 1-807
8	sheep/2016	ISR-2262/3/16	MH816852 1-3944	MH816896 1-2938	MH816860 4-2768	MH816865 1-1948	MH816870 4-1775	MH816895 1-1637	MH816879 8-1105	MH816885 14-1091	MH816888 32-1009	MH816893 4-801
8	cattle/2016	ISR-2475/16	MH816852 4-3942	MH816856 1-2938	MH816861 4-2768	MH816866 1-1946	MH816871 1-1772	MH816875 1-1637	MH816880 7-1105	MH816884 40-1091	MH816890 58-999	MH816894 1-801
8	cattle/2010	1206/10	KF017504 1-3944	JF423934 1-2938	MH816857 1-2767	MH816862 19-1960	MH816867 524-1772	MH816872 1-1635	MH816876 28-1106	MH816881 40-1092	MH816886 31-987	KF584206 1-791
8	sheep/2010	ISR-2089/2/10	KF017506 27-1941	JF423936 1-2938	MK524045 2-1015	MK524046 22-1938	MK524047 77-1294	MK524048 1-1637	MK524049 209-888	MK524050 198-877	JQ970451 41-1000	MK524051 2-812
8	cattle/2010	ISR-1992/10	MT773510 14-814	MT773514 1-2938	MT773520 41-1007	MT773524 37-1059	MT773528 548-1270	MT773533 1-1637	MT773537 41-1032	MT811787 28-1082	MT773544 12-828	MT773549 19-802
8	cattle/2010	ISR-2063/1/10	MT773511 12-811	MT773515 1-2938	MT773521 7-1007	MT773525 46-468	MT773529 549-1269	MT773534 1-1635	MT773538 36-1034	MT773540 71-1078	MT773545 14-921	MT773551 13-806
8	sheep/2010	ISR-2204/10	MT773512 12-817	MT773518 1-2938	MT773522 24-1007	MT773526 37-1052	MT773530 556-1283	MT773536 1-1626	MT773539 38-1042	MT773541 28-1083	MT773546 4-770	MT773552 11-803
8	cattle/2019	ISR-1194/1/19	MT773509 12-819	MT773513 1-2939	MT773519 713-1526	MT773523 193-1105	MT773527 557-1278	MT773532 1-1637	MT811785 280-880	no	MT811786 1-603	MT886701 14-282
8	sheep/2019	ISR-2070/1/19	no	MT773516 37-1382	no	no	no	MT773531 36-932	no	no	no	no
8	sheep/2019	ISR-2075/19	no	MT773517 8-1383	no	no	no	MT773535 40-941	no	no	no	no
16	cattle/2015	ISR-2101/15	MK893157 4-817	KY513456 792-1359	MK893165 102-977	MK893174 211-1062	MK893205 36-1257	no	no	MK893193 53-1009	MK893216 62-976	MK893225 1-812
16	cattle/2013	ISR-2103/5/13	KP306781 1-2851	KP306781 75-2925	KM365247 114-1013	no	MK893204 36-1289	no	no	MK893192 54-1011	MK893215 66-988	MK893224 4-812
16	sheep/2011	ISR-2019/11	KF017517 5-799	KP318798 1000-1728	MK893166 102-992	MK893175 207-1062	MK893206 34-1291	no	MK893202 42-1050	MK893185 49-1008	JQ970461 43-1002	MK893226 1-795
15	cattle/2016	ISR-2455/16	MK893158	MT786704	MK893167	MK893176	MK893203	no	no	MK893186	MK893217	MH048666

			48-814	5-2740	136-988	223-1069	13-1293			55-999	73-979	4-735
12	cattle/2011	ISR-2078/11	KF017510	KT946759	MK893168	MK893177	MK893207	KT946756	MK893196	MK893187	JQ970455	MK893227
			10-816	686-2737	92-967	197-1080	73-1291	1-1640	38-1120	46-987	44-1000	4-812
5	cattle/2015	ISR-1657/2/15	MK893159	MT786703	MK893169	MK893178	MK893208	no	no	MK893188	MK893218	MK893228
			4-814	1-562	110-988	211-1090	36-1292			54-1006	64-987	25-811
5	cattle/2011	ISR-1405/11	MK893160	no	KM365258	MK893179	MK893209	no	no	MK893189	JQ970449	MK893229
			4-811		88-1013	207-1063	36-1290			54-1009	52-1002	4-811
4	sheep/2013	ISR-1899/13	MK893162	MT799822	KM365267	MK893181	MK893211	no	MK893198	MK893190	MK893219	MK893231
			4-816	179-662	88-1013	194-1069	36-1292		30-1043	54-1005	70-986	4-807
4	sheep/2012	ISR-1925/4/12	MK893161	KF584194	MK893170	MK893180	MK893210	no	MK893197	MK893194	MK893223	MK893230
			10-816	174-653	106-990	211-1090	36-1298		40-1033	54-1003	70-987	4-797
4	sheep/2017	ISR-1779/3/17	MK893163	MT793824	MK893171	MK893182	MK893212	no	MK893199	MK893195	MK893220	no
			34-808	20-336	106-994	231-1069	36-1290		33-1044	52-1009	65-987	
2	cattle/2017	ISR-1923/17	MK893164	MT793823	MK893173	MK893184	MK893214	no	MK893201	MK893191	MK893221	MK893233
			4-816	1-368	106-994	209-1069	36-1292		35-1047	55-1009	79-986	4-820
2	sheep/2011	ISR-2054/11	KF017491	no	MK893172	MK893183	MK893213	no	MK893200	no	MK893222	MK893232
			1-798		140-962	202-1050	38-1289		37-1048		79-986	4-811
untyped	sheep/2018	ISR-272/3/18	no	no	no	no	MT396947	no	no	no	no	no
							40-450					
1	sheep/2019	ISR-2070/1/19(1)	no	MT833654	no	no	no	no	no	no	no	no
				1352-2887								
	sheep/2019	ISR-2075/19(1)	no	MT833655	no	no	no	no	no	no	no	no
				1356-2605								

Upper rows provide accession numbers of sequenced regions. Lower rows show sequences regions. Considering close or even 100% identity by most segments ISR2008/13 (sequenced in Pirbright laboratory, United Kingdom) and ISR-1206/10 (sequenced in KVI) its bring us to conclusion that it was the same BTV-8 strain, which was sent to Pirbright for conformation of serotype. The same situation was observed with ISR-1405/11 BTV-5 strain, where it was designated as ISR2011/05.
